# ﻿Taxonomic identity of *Corydalislidenii* (Papaveraceae)

**DOI:** 10.3897/phytokeys.190.80724

**Published:** 2022-02-17

**Authors:** Jun-Tong Chen1, Tian-Hui Kuang, Xian-Han Huang, Xin-Jian Zhang, Hang Sun, Tao Deng

**Affiliations:** 1 CAS Key Laboratory for Plant Diversity and Biogeography of East Asia, Kunming Institute of Botany, Chinese Academy of Sciences, Kunming 650201, Yunnan, China Kunming Institute of Botany, Chinese Academy of Sciences Kunming China; 2 University of Chinese Academy of Sciences, Beijing 100049, China University of Chinese Academy of Sciences Beijing China

**Keywords:** China, *Corydalislidenii*, *Corydalismicroflora*, new synonym, Sichuan, taxonomy

## Abstract

*Corydalismicroflora* and *C.lidenii* are recognised as separate species in “Flora of China” and the latest plant list. However, based on the examination of type specimens and field investigations, *C.lidenii* is shown to be conspecific with *C.microflora*. As a result, *C.lidenii* is synonymised with *C.microflora* in this study.

## ﻿Introduction

The genus *Corydalis* DC., the largest genus of Papaveraceae, contains about 465 species mainly distributed in the northern temperate zone with the highest species diversity in SW China (Wu et al. 1999; Zhang et al. 2008; Chen et al. 2018). Several new species have been described recently, and currently there are about 366 species in this genus known from China. It has been suggested that the uplift of the Qinghai Tibet Plateau and the formation of the Hengduan Mountains, led to an intensive and rapid diversification of this genus (Wang 2006; Sun et al. 2017). The high species diversity of this genus is accompanied by an extremely complex morphology that makes it notoriously difficult for taxonomy and species identification (Wu et al. 1996).

Many species of this genus are known only from type specimens or fewer than 10 specimens. Taking Corydalissect.Trachycarpae (Fedde) Fedde as an example, of 56 species, 17 species are known only from type specimens, 16 species have fewer than 10 specimens, and only 23 species have more than 10 specimens in herbaria. This means that for many species we have a poor understanding of their variation.

*Corydalislidenii* Z.Y.Su is one of those species and was described based on a collection from Maoxishangou, Yusaping, Detuo Town, Luding County, Sichuan Province, China (Su 2008). Su (2008) compared it to *C.pingwuensis* C.Y.Wu, a rather remotely related species. However, it does not seem possible to distinguish *C.lidenii* from the previously described C.flexuosavar.microflora C.Y.Wu in terms of morphology (Zhuang and Wu 1991); and their short rhizomes densely set with fleshy scales are unique in *Corydalis*. After its initial publication, C.flexuosavar.microflora was erected to the subspecific rank as C.flexuosasubsp.microflora (C.Y.Wu & H.Chuang) C.Y.Wu (Wu et al. 1999). Subsequently, Lidén and Su (2007) elevated it to a separate species *C.microflora* (C.Y.Wu & H.Chuang) Z.Y.Su & Lidén. In the account of the family Papaveraceae for “Flora of China” (**FOC**), Zhang et al. (2008) followed Lidén and Su (2007) and treated *C.lidenii* and *C.microflora* as separate species.

**Figure 1.**
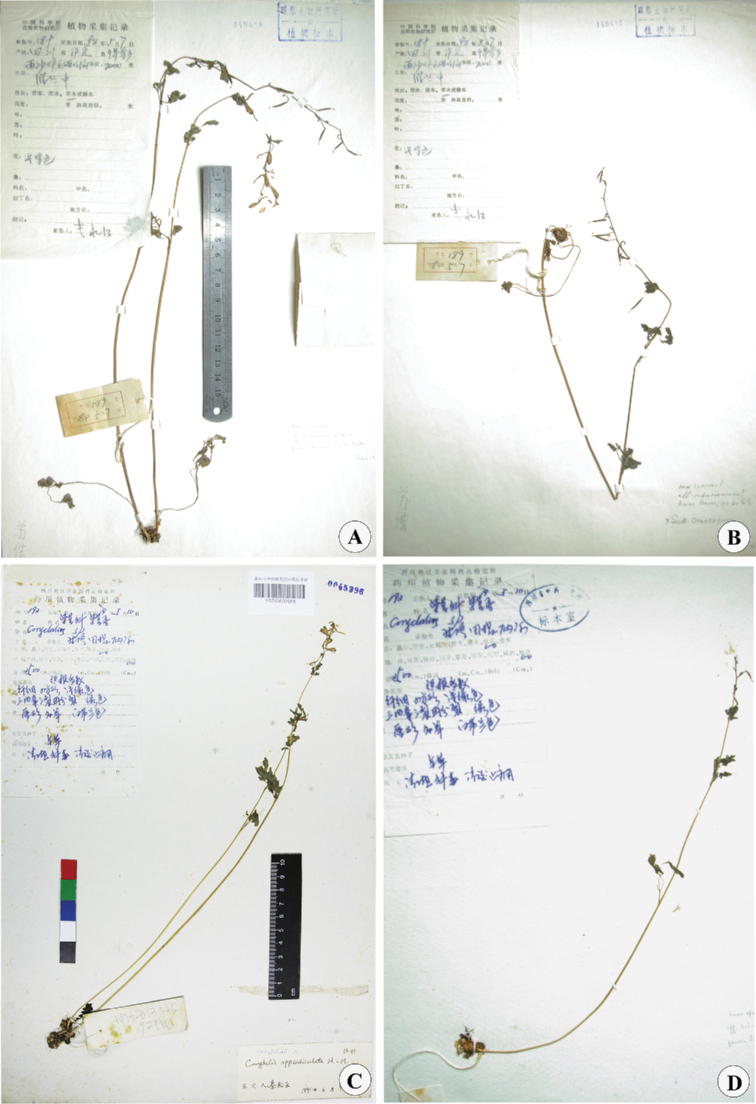
Photographs of the types of *Corydalislidenii***A** lectotype-CDBI149418, designated here! **B** isolectotype-CDBI149417 **C, D** paratypes-SM.

**Figure 2.**
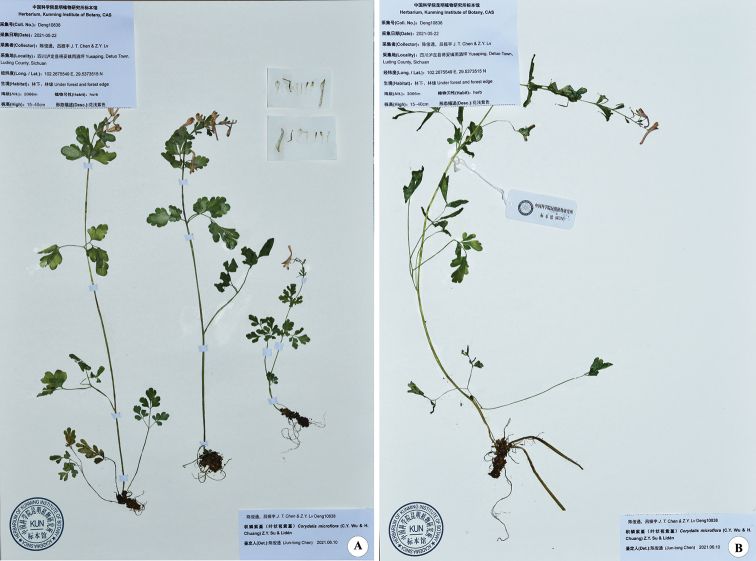
*Corydalismicroflora* from the type locality of *C.lidenii*.

**Figure 3.**
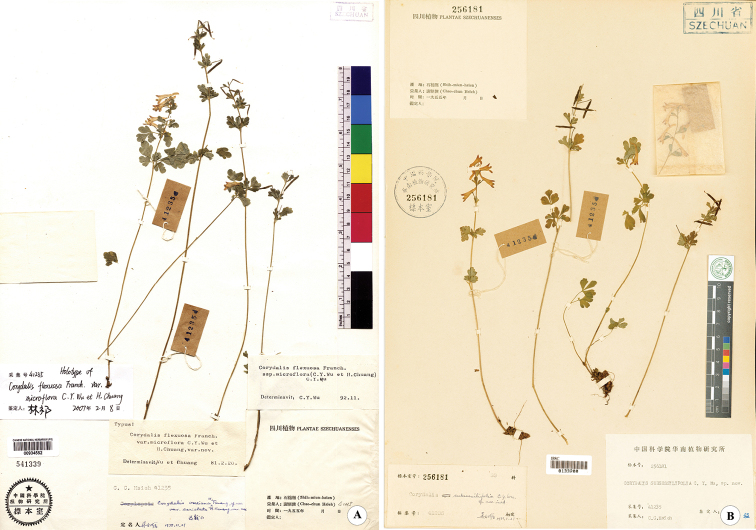
*Corydalismicroflora***A** holotype-PE00934552 **B** isotype-IBSC0133200.

**Figure 4.**
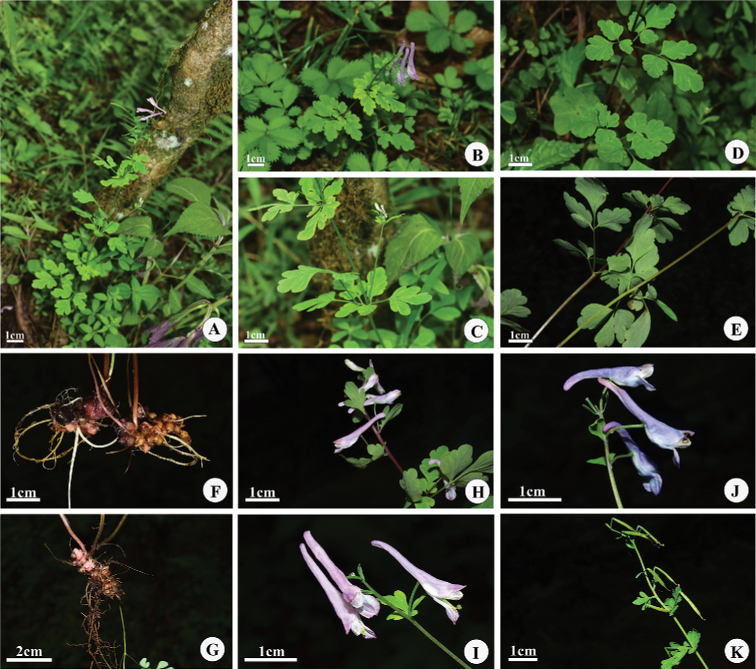
*Corydalismicroflora* at the type locality of *C.lidenii***A** habitat and flowering branch **B** flowering branch **C** small axillary raceme and flower **D** leaf adaxial surfaces **E** leaf abaxial surfaces **F, G** rhizome with small crowded fleshy scales at base **H–J** inflorescence and flowers **K** fruiting raceme.

**Figure 5.**
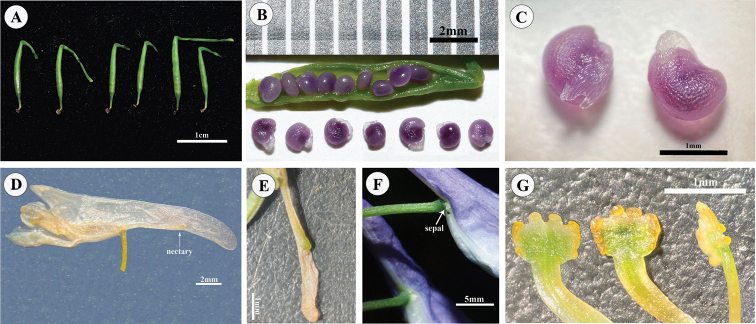
*Corydalismicroflora* at the type locality of *C.lidenii***A** capsule **B** longitudinal section of capsule **C** seeds with elaiosome **D** flower and nectary (arrow) **E** nectary **F** sepal **G** stigma (profile and edge).

**Figure 6.**
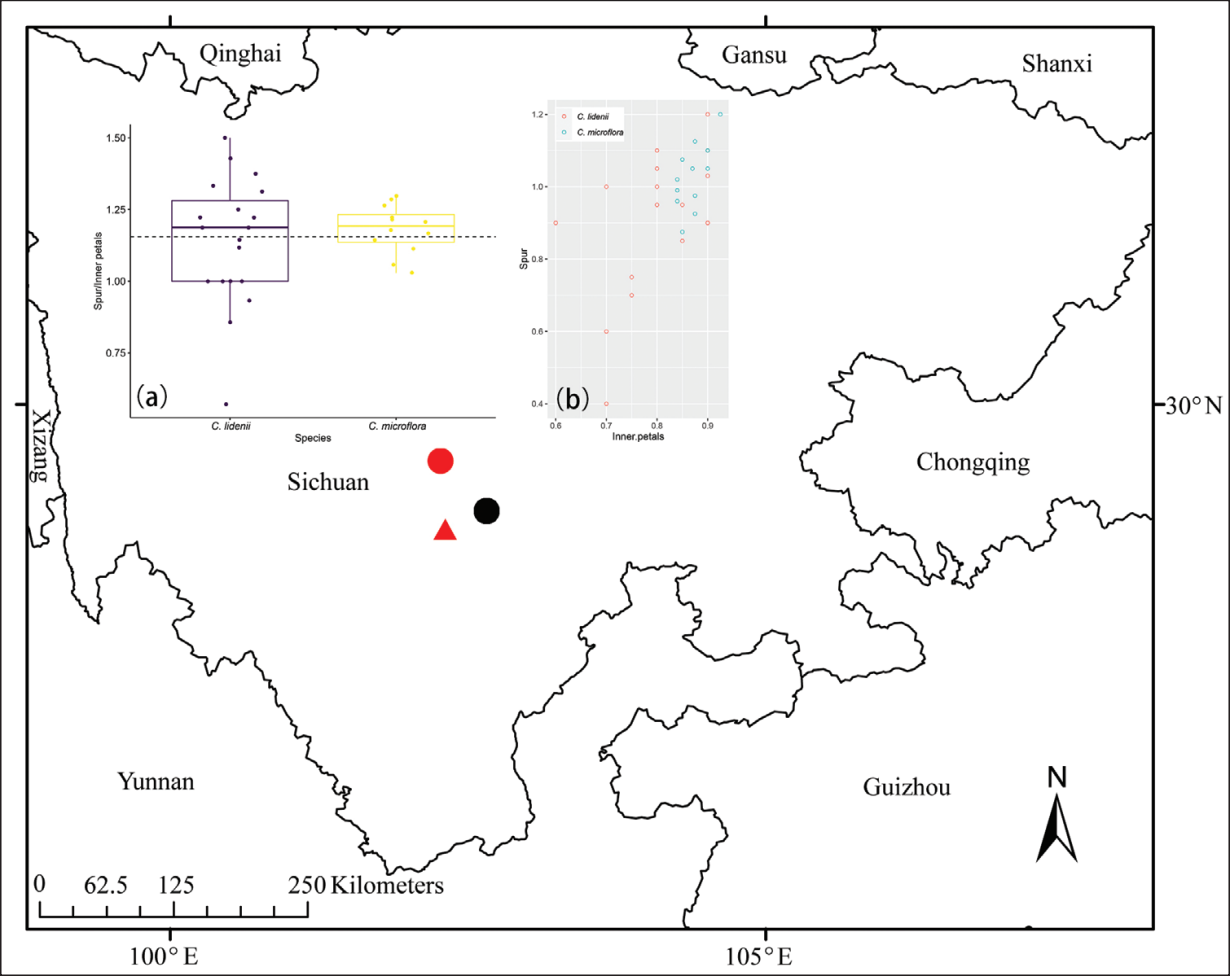
Distribution of *Corydalismicroflora*. (**Notes**: the red dot represents type locality of *C.lidenii* and the black dot represents paratype locality of *C.lidenii*, the red triangle represents the type locality of *C.microflora***a** the Box-plot of comparison of spur/inner petals of *C.lidenii* and *C.microflora***b** the comparison of spur and inner petals of *C.lidenii* and *C.microflora*).

The main differences between *C.lidenii* and *C.microflora* stated by Zhang et al. (2008) are a thin spur longer than the inner petals in the former and a less thin spur equaling the inner petals in the latter. However, when we looked at old and new collections, we noticed that it was not possible to distinguish these two species, and we found no difference in flower and overall morphology between specimens from different localities (Table 1–2; Figs 1–5, 6a, b). Furthermore, the geographical distance between the type localities is very short (Fig. 6). This made us speculate that the two names refer to the same species.

## ﻿Materials and methods

Our recently collected specimens of *Corydalislidenii* are from the type locality: Maoxishangou, Yusaping, Detuo Town, Luding County, Sichuan Province, China. We also studied all specimens of *C.microflora* and *C.lidenii* deposited in the herbaria of PE, CDBI, KUN, SM, IBSC. Additionally, some specimens were obtained through the Chinese Virtual Herbarium (https://www.cvh.ac.cn/). The morphological comparison is provided in Table 1 and Fig. 6.

## ﻿Results and discussion

Based on the detailed morphological comparisons of *Corydalislidenii* and *C.microflora* in rhizome, stems, radical leaves, cauline leaves, inflorescences, flowers and capsule (Table 2), especially the proportion of spur and inner petals (Table 1) emphasized by Zhang et al. (2008), these results show that they are almost identical in overall morphology. Therefore, *C.lidenii* should be treated as a synonym of *C.microflora*.

**Table 1.** Morphological comparisons of *Corydalislidenii* and *C.microflora*.

***C.lidenii***	**1**	**2**	**3**	**4**	**5**	**6**	**7**	**8**	**9**	**10**	**11**	**12**
Spur	0.40	0.60	0.7	0.75	0.85	0.90	0.90	0.95	1.03	0.95	0.95	1.10
Inner petals	0.70	0.70	0.75	0.75	0.85	0.90	0.90	0.85	0.90	0.80	0.80	0.90
***C.lidenii***	**13**	**14**	**15**	**16**	**17**	**18**	**19**					
Spur	1.10	1.00	1.05	1.20	1.10	1.00	0.90					
Inner petals	0.90	0.80	0.80	0.90	0.80	0.70	0.60					
***C.microflora***	**1**	**2**	**3**	**4**	**5**	**6**	**7**	**8**	**9**	**10**	**11**	**12**
Spur	0.88	0.93	0.98	0.96	1.05	0.99	1.05	1.02	1.10	1.08	1.13	1.20
Inner petals	0.85	0.88	0.88	0.84	0.90	0.84	0.87	0.84	0.90	0.85	0.88	0.93

**Notes**: the data of *C.lidenii* are from holotype and specimens collected from its type locality, and numbers 1–4 of *C.lidenii* indicate young flowers; the data of *C.microflora* are from holotype and isotype. Measurement unit: cm.

The following are mainly to modify some taxonomic problems and incorrect records existing in the previous publication of *C.lidenii*, and supplement some type information of *C.microflora*.

### ﻿*Corydalislidenii*

When *C.lidenii* was published, Su (2008) designated *Yong-jiang Li 189* (CDBI) as “holotype”. In herbarium CDBI, two sheets are found and they are not clearly labelled as the parts of a single specimens, and therefore they are not parts of a single specimen but two duplicates of a single gathering according to Art. 8.3 and footnote 1 of *Shenzhen Code* (Turland et al. 2018). Furthermore, Su did not annotate anyone as holotype as such. Therefore, no holotype was actually designated and both of them are syntypes according to Art. 9.5 and Art. 40.2 Note 1. As the specimen CDBI149418 has the anatomical drawing and anatomical records, and the flowers and fruits of this specimen are relatively more complete, it is here selected as lectotype (Fig. 1A, B).

Both specimens of *Yong-jiang Li 189* are in a poor condition; the leaves are folded and damaged and the flowers shrivelled. In May 2021, we therefore went to the type locality (Detuo Town, Luding County) to collect fresh specimens (Fig. 2) of this very delicate species, and found that very careful treatment was required to achieve the perfect condition of the type specimen of *C.microflora*.

Two paratypes were cited in the protologue (Fig. 1C, D). However, the localities for these were recorded incorrectly, and should be Ganluo County, not Dege County. The two counties are quite far apart. This error is repeated by Zhang et al. (2008). The collecting time of the paratypes was misstated to be 1997, but was in fact 1979.

### ﻿*Corydalismicroflora*

Chao-chun Hsieh’s collection records from Shimian County in 1955, show that Hsieh and Xian-xu Kong collected together. Kong is indicated as the collector of only a few specimens, while the others are marked Hsieh. Some collections record different collectors, even on specimens that are obviously duplicates, as they bear the same collection number. There are few complete handwritten collection labels; most are printed labels with incomplete records (only “Shimian County 1955”). We were lucky to find two collection numbers close to the number of the type of *C.microflora* (*C.C.Hsieh 41235*), with more detailed data. One of them, No. 41231 (*Cotoneastermoupinensis* Franch), has two duplicates (PE00501194! and SZ00189464!). The collectors are Kong and Hsieh respectively, but the collection date was 19 June 1955, and a location was beside the highway of Tiezhaizi, Liziping Town, Shimian County, which was recorded in detail on the SZ specimen. Another one No. 41239 (*Valeriana* sp.), has three duplicates (PE01018029!, IBSC0498498! and HGAS029339!). The first two are marked Hsieh whereas the HGAS specimen is marked Kong. The collection date was 20 June 1955 and the locality was Haizishan, Tiezhaizi, Liziping Town, Shimian County (recorded in HGAS029339).

**Table 2.** Comparisons of *Corydalislidenii* and *C.microflora* as given in FOC, and our revised data.

Characters	*C.lidenii* in *FOC*	*C.microflora* in *FOC*	*C.microflora* (Revised)
**Plants**	Herbs, perennial, 25–45 cm tall, glabrous.	Herbs, perennial, 16–33 cm tall, glabrous.	Herbs, perennial, (12–) 16–45 cm tall, glabrous.
**Rhizome**	Rhizome short, with crowded thick pale bulbous (petiolar bases).	Rhizome short, with small crowded fleshy scales or pale callosities at base.	Rhizome short, with small crowded fleshy scales at base.
**Stems**	Stems few, erect, very slender, simple, with 2 leaves in upper 1/2.	Stems 1 to few, erect, slender, simple, with 1 or 2 leaves in upper 1/3–1/2 (possibly also with 1 or 2 early withering small leaves at base).	Stems usually 1 or rarely 2, erect, slender, simple or with 1 tiny flower branch from upper leaf.
**Radical leaves**	Radical leaves early withering, few; petiole 5–9 cm, thin; blade 3–4 × 3–4 cm, thin, bi-(tri-)ternate, with long thin petiolules; leaflets obovate, 5–10 × 3–5 mm, obtuse.	Radical leaves 1 to few with thin petiole 9–12 cm; blade glaucous abaxially, biternate, ca. 3 × 3 cm; leaflets obovate, entire to shallowly 3-lobed.	Radical leaves 0 to 3 with long thin petiole 4–9 cm; blade glaucous abaxially, biternate, ca. 2.5–3 × 2.5–3 cm; leaflets obovate, entire to 3-lobed.
**Cauline leaves**	Cauline leaves shortly stalked or upper sessile, like radical leaves.	Cauline leaves shortly stalked to subsessile, ternate to biternate, 1.5–4 × 2–4 cm; petiolule of lateral primary leaflets 2–5 mm; petiolule of terminal leaflet 5–15 mm; ultimate leaflets broadly obovate, 8–15 mm, ± deeply divided into broad rounded lobes.	Cauline leaves 1–3, like radical leaves; upper leaves shortly stalked or sessile, the lowest leaf usually with long thin petiole.
**Inflorescences**	Racemes very lax, 8–12 cm, 6–8-flowered; lowermost bract leaflike, middle and upper bracts much smaller, oblanceolate, 5–10 mm, entire.	Raceme lax, 5–7- flowered, only slightly elongating in fruit; lower bract like cauline leaf, upper progressively smaller and less divided.	Raceme lax, 4–12 cm, (2–)4–7(–9)- flowered; lower bract like upper cauline leaf, upper progressively smaller and less divided to entire.
**Flowers**	Pedicel ca. 10 mm, 12–20 mm in fruit. Sepal minute. Corolla white to pale blue or pale purple. Outer petals elliptic, acute to shortly mucronate, without crest; upper petal 17–19 mm; spur straight or slightly downcurved, very narrow, ca. 10 mm; nectary ca. 1/2 as long as spur, thin; inner petals ca. 8 mm. Stigma broad, emarginate, basal lobes absent.	Pedicel thin, erect, 6–10 mm in flower, in fruit to 10–15 mm. Sepals 0.5–1 × 0.5–1 mm, large dentate. Corolla probably blue or pale purple. Outer petals subacute, without crest; upper petal 18–19 mm, spur straight or slightly downcurved, narrowly cylindric, 9–10 mm; nectary not recorded; inner petals 9–10 mm. Stigma subcuneate at base (without basal lobes) with 4 marginal apical simple papillae; geminate papillae 1 pair.	Pedicel thin, erect, usually 6–10 mm in flower, lower pedical to 20 mm. Sepal minute, 0.2–0.5 × 0.2–0.5 mm, dentate. Corolla pale purple. Outer petals subacute to shortly mucronate, without crest; upper petal 17–20 mm, spur straight or slightly downcurved, narrowly cylindric, 9–10 mm; nectary ca. 1/2–3/5 as long as spur, thin; inner petals 8–10 mm. Stigma subcuneate at base with (8) 12 papillae.
**Capsule and seed**	Capsule 10–14 × ca. 1 mm, 8–11-seeded; style ca. 2.5 mm. Seeds in 1 row.	Capsule linear, 13–18 mm, 5–13-seeded; style ca. 2.5 mm.	Capsule linear,10–18 × ca. 1 mm, 5–13-seeded; style ca. 2.5 mm. Seeds ca. 1 mm, in 1 row.
**Flowering and fruiting period**	Fl. and fr. May.	Fl. and fr. Jun.	Fl. and fr. May–Jun.
**Distribution**	Sichuan (Luding, Ganluo).	Sichuan (Shimian).	Sichuan (Shimian, Luding, Ganluo).

The collection date of the type of *C.microflora* should be 19 or 20 June 1955, and the locality should be Tiezhaizi to Haizishan, Liziping Town, Shimian County. The distance between this location and that of the type of *C.lidenii* is ca. 70 km, and the distance to the location of the paratypes is ca. 40 km (Fig. 6).

### ﻿Taxonomic treatment

#### ﻿ Corydalis microflora

Taxon classificationPlantaeRanunculalesPapaveraceae

(C.Y.Wu & H.Chuang) Z.Y.Su & Lidén, Novon 17(4): 484. (Lidén and Su 2007)

BB5C8BFC-23A9-5B38-9F9F-B127559B5D73

≡ Corydalisflexuosavar.microflora C.Y.Wu & H.Chuang, Acta Bot. Yunnan.13(2): 132 (Zhuang and Wu 1991); C.flexuosasubsp.microflora (C.Y.Wu & H.Chuang) C.Y.Wu, Fl. Reipubl. Popularis Sin. 32: 118 (Wu et al. 1999). – Type: China. Sichuan: Shimian County, Liziping Town, Tiezhaizi to Haizibian, June 1955, *C.C. Hsieh* & *X.X. Kong 41235* (holotype: PE00934552!, isotype: IBSC0133200!)
= Corydalislidenii Z.Y.Su, Acta Bot. Yunnan. 30(4): 422 (Su 2008), syn. nov. – Type: China. Sichuan: Luding County, Detuo Town, Yusaping, Maoxishangou, 2,000 m, in shrubs, 7 May 1984, *Y.J. Li 189* (lectotype: CDBI149418!, designated here; isolectotype, CDBI149417!)

##### Description.

Herbs, perennial, (12–) 16–45 cm tall, glabrous. Rhizome short, with small crowded fleshy scales at base. Stems usually 1 or rarely 2, erect, slender, simple or with 1 tiny flower branch from upper leaf. Radical leaves 0 to 3 with long thin petiole 4–9 cm; blade glaucous abaxially, biternate, ca. 2.5–3 × 2.5–3 cm; leaflets obovate, entire to 3-lobed. Cauline leaves 1–3, like radical leaves; upper leaves shortly stalked or sessile, the lowest leaf usually with long thin petiole. Raceme lax, 4–12 cm, (2–)4–7(–9)- flowered; lower bract like upper cauline leaf, upper progressively smaller and less divided to entire. Pedicel thin, erect, usually 6–10 mm in flower, lower pedical to 20 mm. Sepals minute, 0.2–0.5 × 0.2–0.5 mm, dentate. Corolla pale purple. Outer petals subacute to shortly mucronate, without crest; upper petal 17–20 mm, spur straight or slightly downcurved, narrowly cylindric, 9–10 mm; nectary 1/2–3/5 as long as spur, thin; inner petals 8–10 mm. Stigma subcuneate at base with (8) 12 papillae. Capsule linear, 10–18 × ca. 1 mm, 5–13-seeded; style ca. 2.5 mm. Seeds ca. 1 mm, in 1 row.

##### Phenology.

Flowering and fruiting from May–June.

##### Distribution and habitat.

*Corydalismicroflora* is a rare species with a narrow distribution in Sichuan, China (Shimian County, Luding County and Ganluo County). It grows in forest margins, open forest, or near valley stream at an elevation of 2,000–2,500 m. Associated species include *Betula* sp. (Betulaceae), *Acer* sp. (Sapindaceae), *Rodgersiaaesculifolia* Batalin (Saxifragaceae), *Paris* sp. (*Melanthiaceae*), *Veronicasutchuenensis* Franch. (Plantaginaceae), *Campylandra* sp. and *Ophiopogon* sp. (Asparagaceae), *Elatostema* sp. (Urticaceae), *Calanthetricarinata* Lindl. (Orchidaceae), *Mimulusszechuanensis* Y.Y.Pai (Phrymaceae), *Corydalisdavidii* Franch. and *Ichtyoselmismacrantha* (Oliver) Lidén (Papaveraceae), amongst others.

##### Additional specimens examined.

– China. **Sichuan**: Ganluo County, Tianba Town to Lianghe Town, elev. ca. 2,500 m, 10 May 1979, *Xichang institute of drug control 192* (SM); Luding County, Detuo Town, Yusaping, 29.53735N, 102.26755E, elev. ca. 2,060 m, under forests and forest margins, 22 May 2021, *J.T.Chen & Z.Y.Lv Deng10838* (KUN).

##### Conservation status.

At present, *Corydalismicroflora* has been found only in three places in Sichuan (Fig. 6), and only four specimens of this species have been collected so far. During a field investigation in 2021, we once again visited the Yusaping, Maoxishangou in Luding. Among four ravines investigated, this species was only found in one. The population was small, ca. 30 individuals were observed, with the extent of occurrence of ca. 1 km^2^. It was growing scattered in open deciduous broad-leaved forests near the mouth of the ravine. Further studies are needed to assess its conservation status, and we only temporarily assign it to the category ‘Data Deficient’ (DD) of the International Union for Conservation of Nature (IUCN 2019).

## Supplementary Material

XML Treatment for Corydalis microflora

## References

[B1] Chen YS Deng T Zhou Z Sun H (2018) Is the east Asian flora ancient or not? National Science Review 5(6): 142–154. 10.1093/nsr/nwx156

[B2] IUCN (2019) Guidelines for Using the IUCN Red List Categories and Criteria. version 13. Prepared by the Standards and Petitions Subcommittee of the IUCN Species Survival Commission, 113 pp. http://cmsdocs.s3.amazonaws.com/RedListGuidelines.pdf

[B3] Lidén M Su ZY (2007) New Species of *Corydalis* (Fumariaceae) from China II. Novon 17(4): 479–496. 10.3417/1055-3177(2007)17[479:NSOCFF]2.0.CO;2

[B4] Su ZY (2008) A New Species of *Corydalis* (Fumariaceae) from China. Yunnan Zhi Wu Yan Jiu 30(4): 422. 10.3724/SP.J.1143.2008.00422 [in Chinese]

[B5] Sun H Zhang JW Deng T Boufford DE (2017) Origins and evolution of plant diversity in the Hengduan Mountains, China. Plant Diversity 39(4): 161–166. 10.1016/j.pld.2017.09.004 30159507 PMC6112316

[B6] Turland NJ Wiersema JH Barrie FR Greuter W Hawksworth DL Herendeen PS Knapp S Kusber WH Li DZ Marhold K May TW McNeill J Monro AM Prado J Price MJ Smith GF (2018) International Code of Nomenclature for algae, fungi, and plants (Shenzhen Code) adopted by the Nineteenth International Botanical Congress Shenzhen, China, July 2017. Regnum Vegetabile 159. Koeltz Botanical Books, Glashütten. 10.12705/Code.2018

[B7] Wang YW (2006) Study on the phylogenetic of *Corydalis*. PhD Thesis, Institute of Botany, the Chinese Academy of Sciences, China. [in Chinese]

[B8] Wu ZY Zhuang X Su ZY (1996) The systematic evolution of *Corydalis* in relation to florogenesis and floristic regionalization in the world. Yunnan Zhi Wu Yan Jiu 18(3): 241–267. [in Chinese]

[B9] Wu ZY Zhuang X Su ZY (1999) *Corydalis* DC. In: Wu ZY (Ed.) Flora Reipublicae Popularis Sinicae, vol.32. Science Press, Beijing, 96–481. [in Chinese]

[B10] Zhang ML Su ZY Lidén M (2008) *Corydalis* DC. In: Wu ZY Raven PH Hong DY (Eds) Flora of China, Volume 7.Science Press, Beijing and Missouri Botanical Garden Press, St. Louis, 295–428.

[B11] Zhuang X Wu ZY (1991) The Classification and Distribution of Chinese Corydalissect.Asterostigmata. Yunnan Zhi Wu Yan Jiu 13(2): 132. [in Chinese]

